# Commentary: Association between composite dietary antioxidant index and increased urinary albumin excretion: a population-based study

**DOI:** 10.3389/fnut.2025.1667532

**Published:** 2025-12-03

**Authors:** Xue-Feng Jin, Wen-Hui Tong, Jing-Ping Ge, Tong Zhang

**Affiliations:** 1 Organ Transplantation Clinical Medical Center of Xiamen University, Department of General Surgery, Xiang'an Hospital of Xiamen University, School of Medicine, Xiamen University, Xiamen, China; 2 Organ Transplantation Institute of Xiamen University, Xiamen Human Organ Transplantation Quality Control Center, Xiamen Key Laboratory of Regeneration Medicine, Fujian Provincial Key Laboratory of Organ and Tissue Regeneration, School of Medicine, Xiamen University, Xiamen, China; 3 Medical College of Yangzhou University, Yangzhou, Jiangsu, China; 4 Department of Urology, Affiliated Taikang Xianlin Drum Tower Hospital, Medical School of Nanjing University, Nanjing, Jiangsu, China; 5 Department of Urology, Taikang Xianlin Drum Tower Hospital Clinical College of Wuhan University, Nanjing, Jiangsu, China

**Keywords:** composite dietary antioxidant index, urinary albumin excretion (UAE), dietary antioxidants, albuminuria, NHANES

In their recent article published in Frontiers in Nutrition, Li et al. explored the association between the Composite Dietary Antioxidant Index (CDAI) and urinary albumin excretion using NHANES 2007–2018 data. While the topic is highly relevant, we would like to offer several methodological clarifications and suggestions for improving transparency and reproducibility.

In the article, the authors reported a sample size of 70,190 participants for the period 2007–2018. However, according to the official NHANES documentation, each two-year survey cycle includes approximately 10,000 participants (1). Therefore, the total number of participants across the six survey cycles from 2007 to 2018 should be around 60,000. Through our analysis of the NHANES database from 2007 to 2018, we obtained the following participant numbers for each survey cycle: 2007–2008: 10,149 participants; 2009–2010: 10,537 participants; 2011–2012: 9,756 participants; 2013–2014: 10,175 participants; 2015–2016: 9,971 participants; 2017–2018: 9,254 participants. In total, the combined number of participants across all six cycles is 59,842. Moreover, the participant numbers reported in numerous published studies based on NHANES 2007–2018 data are consistent with the figures we calculated (2–4).

In addition, the calculation method for the Composite Dietary Antioxidant Index (CDAI) is only briefly described by referencing the study by Wright et al., without providing the actual formula or specific examples. To ensure reproducibility and facilitate secondary analysis, we recommend providing a detailed description of the CDAI calculation method.

Although the participant selection process is outlined in Figure 1, there is a lack of detailed explanation regarding the number of exclusions based on each covariate. Adding clarification on these figures would enhance the transparency and credibility of the article. Moreover, given the complex survey design of NHANES, weighted analysis is essential to ensure the national representativeness of the results. Clearly stating whether sampling weights were applied would strengthen the validity of the study.

Finally, we noticed that the term “microalbuminuria” is used in the introduction. According to the 2021 KDIGO guidelines, this term is now considered outdated and should be replaced with “moderately increased albuminuria” to ensure consistency and accuracy with current clinical standards.

In summary, although the study by Li et al. provides valuable insights, addressing the issues mentioned above would help enhance the transparency, reproducibility, and clinical relevance of the research.
